# Systematic identification and analysis of WRKY transcription factors reveals the role of MrWRKY14 in *Myrica rubra*

**DOI:** 10.3389/fpls.2025.1602750

**Published:** 2025-05-30

**Authors:** Xiurun Fan, Minghui Chen, Huiling Zhang, Yumeng Liu, Meng Yang, Chengyang Ye, Hailing Gu, Kai Xu, Boping Wu

**Affiliations:** Collaborative Innovation Center for Efficient and Green Production of Agriculture in Mountainous Areas of Zhejiang Province, College of Horticulture Science, Zhejiang A&F University, Hangzhou, Zhejiang, China

**Keywords:** bayberry, WRKY gene family, phylogenetic tree, collinearity, expression pattern analysis

## Abstract

Bayberry (*Myrica rubra*) is a significant subtropical fruit tree, renowned for its distinctive flavor and high nutritional value. WRKY transcription factors are a class of plant-specific zinc-finger proteins that play critical roles in plant growth and development, secondary metabolism, and responses to abiotic stress. However, there is currently limited information about the *WRKY* gene family in bayberry. This study conducted a systematic bioinformatics analysis of 55 *WRKY* genes in bayberry, elucidating their phylogenetic relationships, gene structures, conserved motifs, and syntenic characteristics. The results demonstrated that these *WRKY* family members could be classified into five subfamilies, with each gene containing at least one WRKY domain. The bayberry *WRKY* genes exhibited significant variations in gene length and intron-exon numbers, while maintaining relatively conserved gene structures within each subfamily. The promoters of *WRKY* gene members contained multiple regulatory elements, including hormone-responsive elements, light-responsive elements, and abiotic stress-responsive elements. Collinearity analysis revealed that the *WRKY* family in bayberry experienced six segmental duplication events. Inter-species synteny analysis demonstrated high collinearity between bayberry and *Actinidia* spp., indicating evolutionary conservation of *WRKY* genes across different plant species. It was observed that bayberry *WRKY* genes exhibited significant differential expression across different cultivars and developmental stages of fruits through expression pattern analysis. Further research indicated that MrWRKY14, a member of the bayberry WRKY family, significantly enhanced the promoter activity of *MrSWEET1*, thereby influencing the process of sugar accumulation. These findings not only provide an important reference for the genome-wide identification of *WRKY* gene families in plants but also lay a solid foundation for future in-depth functional analysis of bayberry *WRKY* genes.

## 1 Introduction

WRKY transcription factor family is one of the largest transcription factor families in higher plants and has been found throughout the green plant lineage (Ulker and Somssich, 2004). The name of “WRKY family” derived from its highly conserved 60-amino acid, four-stranded β-sheet WRKY DNA-binding domain (DBD), which contained the highly conserved N-terminal WRKYGQK motif and a C-terminal zinc finger motif with novel metal-chelating properties (Zhang and Wang, 2005). Methionine and histidine residues coordinated a zinc ion to form a finger-like structure motif. Both the WRKY domain and zinc finger motif were essential for DNA binding activity of the protein (Yang et al., 2009). Based on these features, the WRKY family members were classified into three groups: Group I (with two WRKY DBDs), Group II (with a single DBD and diverse C2H2 zinc finger structures), and Group III (with a single DBD and C2HC zinc finger structures) (Rushton et al., 2010). Group I *WRKY* genes were characterized by dual WRKY domains, whereas Group II and III *WRKY* genes possessed only a single domain (Maeo et al., 2001). Structurally, both Group I and II contained a C2H2-type zinc finger motif (C-X4-5-C-X22-23-H-X1-H), with DNA-binding activity exclusively mediated by the C-terminal domain. Group II WRKY proteins were further subdivided into five subgroups (a-e) based on variations in additional amino acid motifs outside the WRKY domain. Notably, Group III exhibited significant divergence in zinc finger configuration compared to Groups I and II, featuring a distinctive C2-HC zinc finger structure with a unique C-X7-C-X23-H-X-C arrangement pattern (Bakshi and Oelmüller, 2014).

WRKY protein family has been extensively identified across diverse plant species, exhibiting significant interspecies variation in gene family size. Notable examples included the identification of 65 members in *Arabidopsis thaliana* (Wang et al., 2011), 101 members in rice (*Oryza sativa*) (Abdullah-Zawawi et al., 2021), and 72 members in tomato (*Solanum lycopersicum*) (Liu et al., 2022). In fruit tree species, the distribution of WRKY family members exhibited a more pronounced diversity and abundance. For instance, 113 members have been identified in apples (Qin et al., 2022), 116 members in kiwifruit (Jing and Liu, 2018), 47 members in sweet orange (Xi et al., 2023), and 59 in grape (Wang et al., 2014).

Extensive research has confirmed that WRKY transcription factors play crucial biological roles in plants, primarily in regulating plant dwarfism, leaf senescence, fruit ripening, and responses to abiotic stress. For instance, studies in apple have revealed that the transcription factor MdWRKY9 promoted dwarfism by directly suppressing the transcriptional activity of MdDWF4, a key enzyme involved in brassinosteroid (BR) biosynthesis, thereby reducing BR production (Zheng et al., 2018). In strawberry, FaWRKY transcription factors FaWRKY48, FaWRKY53, FaWRKY24 and FaWRKY17 were involved in the abscisic acid signaling pathway, promoting fruit ripening by regulating ABA biosynthesis (Garrido-Gala et al., 2022). Additionally, the expression of *WRKY* gene were induced by thyme chitosan nanoparticles, thereby enhancing tomato resistance to root rot pathogens. The expression of *WRKY* genes has been demonstrated to enhance plant drought tolerance while simultaneously promoting plant growth and biomass accumulation under drought conditions. For instance, *TaWRKY133* functioned as a negative regulator of drought stress responses in wheat. Overexpression of *TaWRKY133* reduced the drought tolerance of transgenic plants, highlighting its critical role in regulating abiotic stress responses (Lv et al., 2022). Overexpression of *AtWRKY30* has been demonstrated to improve drought tolerance in transgenic wheat by upregulating the expression of genes associated with growth, osmoregulatory substance biosynthesis, gas exchange parameters, and antioxidant enzyme activity (Yang et al., 2025).

Multiple studies have demonstrated that WRKY transcription factors play crucial roles in fruit development, ripening, and quality formation. In bananas, approximately 50% of the *MaWRKY* gene family members exhibited high expression during fruit ripening, indicating their significant regulatory roles in fruit development and post-harvest ripening processes (Jia et al., 2022). Research on strawberries has shown that *FaWRKY71* expression was induced by exogenous abscisic acid, but it does not affect endogenous ABA synthesis. Instead, *FaWRKY71* promoted strawberry fruit ripening through auxin regulation rather than the ABA pathway (Yue et al., 2022). In pitaya, HpWRKY3 activated the expression of *HpINV2* and *HpSuSy1*, indicating that this gene may participate in sugar accumulation in dragon fruits by transcriptionally regulating sucrose metabolism-related genes (Wei et al., 2019). In grapes, overexpression of *VvWRKY22* reduced the content of sucrose, glucose, and fructose, modulated the expression of genes related to sugars and ABA, and interacted with VvSnRK1.1 or VvSnRK1.2 proteins (sucrose non-fermenting-1 related protein kinases), which were important kinases involved in sugar metabolism (Huang et al., 2021).

Bayberry (*Myrica rubra*) is an important subtropical fruit tree native to China (Sun et al., 2013). WRKY transcription factors played crucial regulatory roles in plant growth and development, secondary metabolism, and responses to abiotic stress (Rushton et al., 2010). However, current research on the *WRKY* gene family in bayberry remained relatively limited. In this study, we conducted a comprehensive analysis of the *WRKY* gene family in bayberry fruits through genome-wide identification and expression pattern analysis, aiming to establish a solid theoretical foundation for elucidating their biological functions. These findings were expected to provide valuable references for elucidating the potential applications and molecular mechanisms of *WRKY* gene family in plants, thereby supporting their further development and utilization.

## 2 Materials and methods

### 2.1 Plant materials

Fruits of two bayberry cultivars (‘Dongkui’ and ‘Shuijing’) were selected as experimental materials, collected from LinGe Family Ecological Farm in Lin’an District, Hangzhou City, Zhejiang Province. Fruit samples were collected at five different developmental stages (designated S1-S5), corresponding to 51, 58, 65, 72, and 80 days after flowering, respectively. The experiment was conducted with three biological replicates, each containing at least five fruits. All collected samples were immediately frozen in liquid nitrogen and stored at -80°C for subsequent analysis.

### 2.2 Identification of *WRKY* gene family members in bayberry

To screen the *WRKY* genes of bayberry, the genome sequences and annotation information of bayberry was downloaded from the NCBI database (https://www.ncbi.nlm.nih.gov/datasets/genome/GCA_003952965.2/). The WRKY sequences of *Arabidopsis* were obtained from The Arabidopsis Information Resource (TAIR) (https://www.arabidopsis.org/). The hidden Markov model (HMM) for the WRKY conserved domain (PF03106) was retrieved from the Pfam protein family database. Using this HMM model and the *Arabidopsis* WRKY protein sequences, candidate *WRKY* genes in bayberry was identified through HMMER and BLASTP tools. Initially, the *Arabidopsis* sequences were used as query sequences to perform a BLASTP search against the bayberry proteome (e-value < 1e-10, identity >= 40). The WRKY proteins in the bayberry genome was identified using the HMM search function in TBtools, and redundant sequences were removed from the results. Molecular weight (Da), isoelectric points, and other physicochemical properties of WRKY proteins were determined using the online ExPASy tool (http://web.expasy.org/protparam/).

### 2.3 Phylogenetic tree analysis of *WRKY* genes

A Clustal X2 sequence alignment was performed on the full-length protein sequences of *Arabidopsis* and bayberry WRKY proteins. The phylogenetic tree was constructed using the MEGA X software with the Neighbor-Joining method, and bootstrap resampling was set to 1000 repetitions. The phylogenetic tree was subsequently polished using the ITOL online tool (https://itol.embl.de/).

### 2.4 Gene structure and conserved motifs analysis

The exon and intron information of bayberry *WRKY* genes was retrieved from the bayberry genome annotation data. The gene structure diagram was generated using the Visualiza Gene Structure function in TBtools. For motif analysis, the MEME suite (http://meme-suite.org/tools/meme) was employed to identify six conserved motifs within the bayberry WRKY protein sequences. The distribution of these motifs was visualized using the Gene Structure View function in TBtools.

### 2.5 Chromosome location and collinearity analysis

The chromosome positions and tandem repeats of *MrWRKY* genes were visualized through the BLAST and MCScanX functions in TBtools software based on the MrWRKY protein sequences. Based on the bayberry genome and annotation files, whole-genome sequence alignment was conducted through the MCScanX and gene position extract functions in TBtools, yielding syntenic relationship files of *MrWRKYs* within the bayberry species. The chromosome length file was obtained using the fasta stats function, while the gene density file was generated via the fasta stats table function. Finally, by integrating the syntenic relationship file, chromosome length file, and gene density file, the homologous gene diagram for *MrWRKYs* were constructed using the Advanced Circos function zone of TBtools software.

### 2.6 *Cis*-acting elements analysis

The *cis*-acting elements in the 2000 bp promoter regions of each bayberry *WRKY* gene family members were analyzed using the PlantCARE website (https://bioinformatics.psb.ugent.be/webtools/plantcare/html/). And the analysis results were visualized using the TBtools software.

### 2.7 Gene expression profile analysis

Total RNA was extracted from various cultivars of bayberry during different fruit development stages using the CTAB method (Liu et al., 2024). RNA quantity and quality were determined using a NanoDrop One spectrophotometer, and RNA integrity was assessed by 1% agarose gel electrophoresis. High-quality cDNA libraries were constructed, and gene expression analysis was performed. A transcriptome-wide expression profile of bayberry *WRKY* genes was generated using strand-specific RNA-seq, with transcript abundance calculated as reads per kilobase of transcript per million mapped reads (RPKM). Sequencing data used in the current study are available in the NCBI Sequence Read Archive database (project number: PRJNA1105392). Expression patterns of bayberry *WRKY* genes were visualized as a heatmap using the Heatmap illustrator tool in TBtools. The relative expression levels of genes were calculated using the 2^−ΔΔCt^ method, with *Mr-actin* serving as the reference gene for normalization. All experiments were conducted with three biological and technical replicates to ensure reproducibility.

### 2.8 Dual luciferase analysis

The promoter sequence was inserted upstream of the firefly luciferase (LUC) reporter gene vector, while the Renilla luciferase (REN) gene, driven by the 35S promoter, served as an internal control on the same vector. The coding sequence (CDS) of MrWRKY was cloned into the pGreenII 0029 62-SK vector as the effector gene, with the empty vector serving as the negative control. For transient expression assays, Agrobacterium tumefaciens strains harboring either the effector or reporter constructs were co-infiltrated into the abaxial side of Nicotiana benthamiana leaves using Agrobacterium-mediated transformation. After 48 hours of incubation, luciferase activity was measured using a dual-luciferase reporter assay system. The relative LUC/REN ratio was calculated according to the instructions of the dual luciferase reporter gene assay kit.

### 2.9 Statistical analysis

Data were processed, statistically analyzed and calculated using Microsoft Excel software. Data visualization was performed with OriginPro 2023. Independent two-sample t-tests were performed using IBM SPSS Statistics 27.0, with *P*<0.05 considered statistically significant. Gene expression profiles were compared using Pearson correlation analysis.

## 3 Results

### 3.1 Identification and phylogenetic analysis of bayberry *WRKY* genes

By integrating the hidden Markov model (HMM) and BLAST search, a total of 55 *WRKY* gene family members were identified from the bayberry genome data, and were systematically designated as *MrWRKY01* to *MrWRKY55* based on their chromosomal locations. The MrWRKY proteins exhibited significant variations in their physicochemical properties, including amino acid length and theoretical isoelectric point values (**Supplementary Table 1**). Specifically, the amino acid numbers of MrWRKYs varied from 131aa (MrWRKY02) to 990 aa (MrWRKY45), the molecular weights ranged from 14879.22 Da (MrWRKY02) to 108143.45 Da (MrWRKY02). And the pI values fluctuated between 4.94 (MrWRKY30) and 9.89 (MrWRKY48). In addition, 42 (76.36%) members of the MrWRKYs were localized in the nuclear, 8 (14.54%) members were localized in the cytoplasmic, 3 (5.45%) members were localized in the chloroplast.

**Figure 1:**
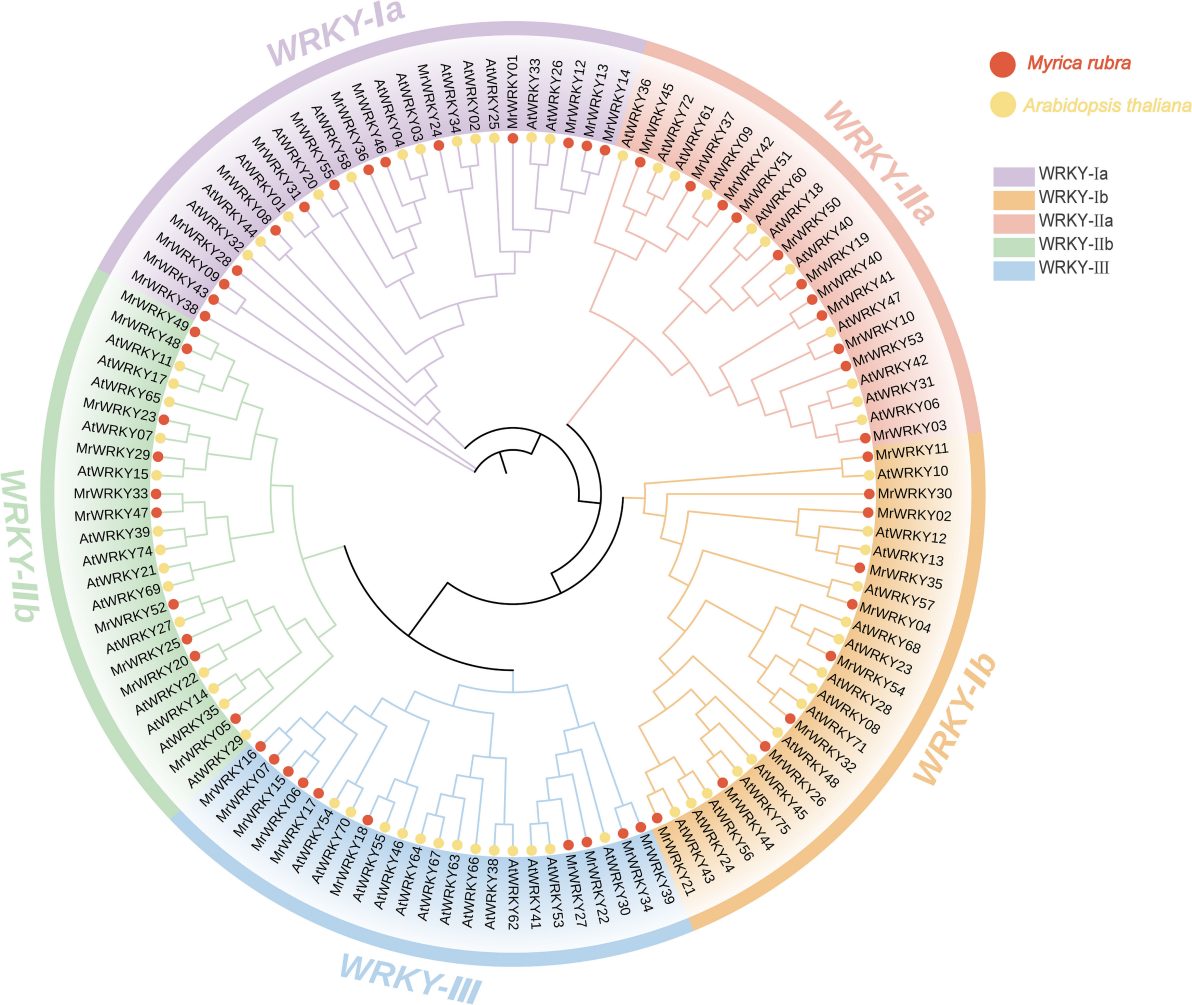
Phylogenetic analysis of WRKY proteins in *Myrica rubra* and *Arabidopsis thaliana*. Red dots correspond to *MrWRKY* genes from bayberry, while yellow dots represent *AtWRKY* genes from *Arabidopsis*. The different subfamilies are represented by distinct colored blocks for visual differentiation.

To comprehensively understand the evolutionary relationships and functional conservation of the *WRKY* genes, multiple sequence alignments were performed on WRKY protein sequences from bayberry and *Arabidopsis*. As shown in **Figure 1**, a phylogenetic tree was constructed using the Neighbor-Joining (NJ) method. The *WRKY* genes of bayberry were systematically classified following the established classification scheme of *Arabidopsis WRKY* genes. The results showed that bayberry *WRKY* genes could be divided into five subfamilies: WRKY-Ia, WRKY-Ib, WRKY-IIa, WRKY-IIb, and WRKY-III. Among them, WRKY-Ia subfamily comprised 14 genes, representing the largest subgroup within the *MrWRKY* gene family, suggesting its highest level of diversity and complexity. In contrast, the WRKY-IIa and WRKY-IIb subfamilies each contained 11 genes, reflecting distinct evolutionary trajectories and adaptive capabilities among WRKY subfamilies. The WRKY-Ib and WRKY-III subfamilies, each consisting of 10 genes, constitute the smallest subgroups, and these quantitative variations likely reflect differential selection pressures and retention mechanisms during evolutionary processes.

### 3.2 Chromosome location analysis of bayberry *WRKY* genes

**Figure 2:**
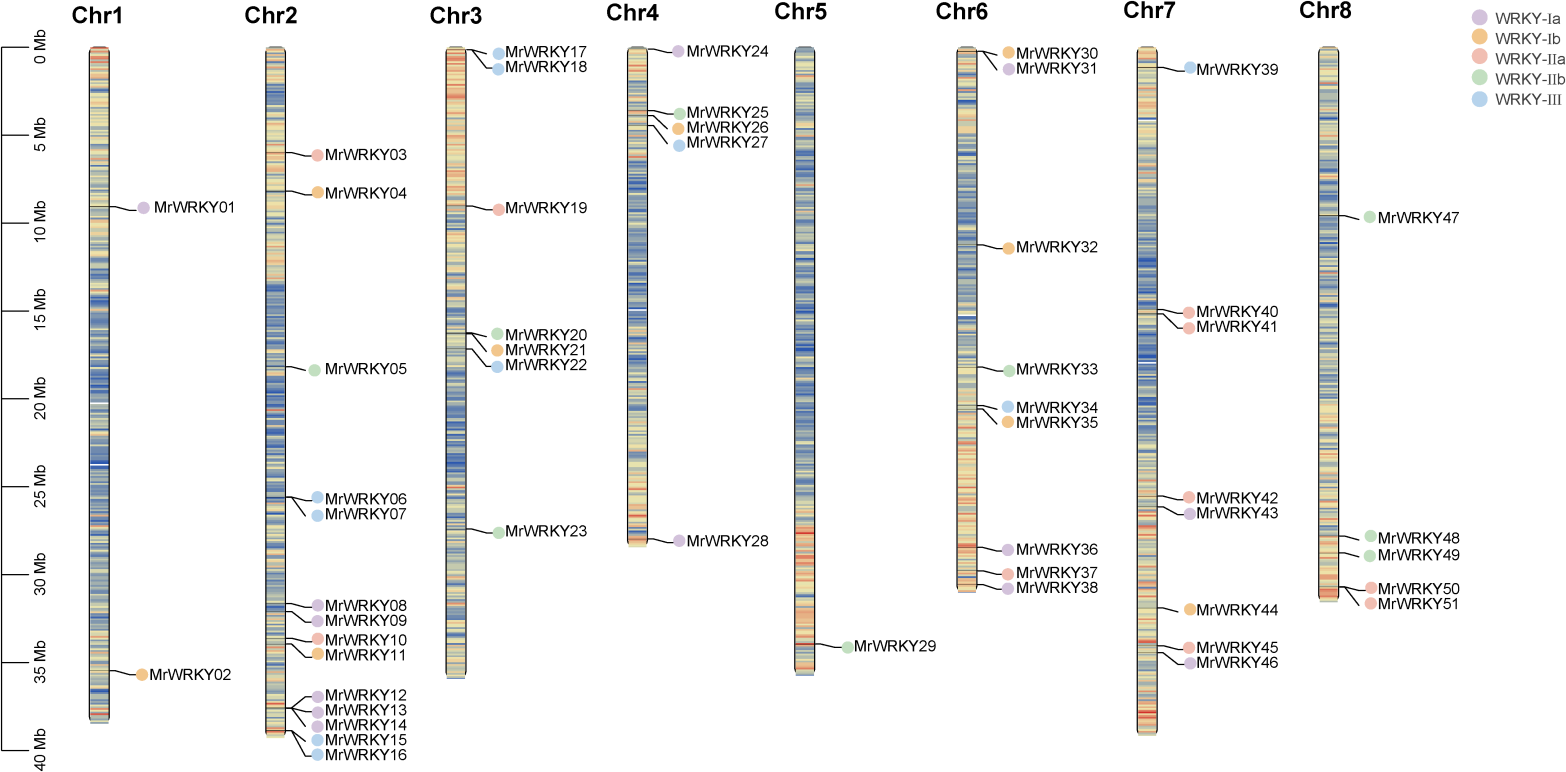
Chromosomal distribution of *WRKY* genes in bayberry. Genes from different subfamilies are represented by distinct colored dots.

The chromosome location analysis of bayberry WRKY genes were conducted by the Tbtools tool. As shown in **Figure 2**, 51 *MrWRKY* genes were mapped across the eight chromosomes of bayberry, while four *MrWRKY* genes (*MrWRKY52*, *MrWRKY53*, *MrWRKY54*, and *MrWRKY55*) were localized on the scaffold. These 51 *MrWRKY* genes were unevenly distributed across eight chromosomes. Notably, Chromosome 2 contained the highest number of *MrWRKY* genes (14 members), while chromosomes 1, 3, 4, 6, 7, and 8 harbored 2, 7, 5, 9, 8, and 5 genes, respectively. Strikingly, only a single *MrWRKY* gene was found on chromosome 5. This uneven genomic distribution pattern suggested that the chromosomal allocation of *WRKY* genes may be associated with structural and functional characteristics of the respective chromosomes.

Interestingly, chromosomes 2 and 5 exhibited the most abundant distribution of *WRKY* gene subfamilies, encompassing WRKY-Ia, WRKY-Ib, WRKY-IIa, WRKY-IIb, and WRKY-III subfamilies. Considering the pivotal role of chromosome scaffolds in gene expression and regulation, these scaffold-associated *WRKY* genes may confer distinctive biological functions.

### 3.3 Gene structure analysis of bayberry *WRKY* genes

**Figure 3:**
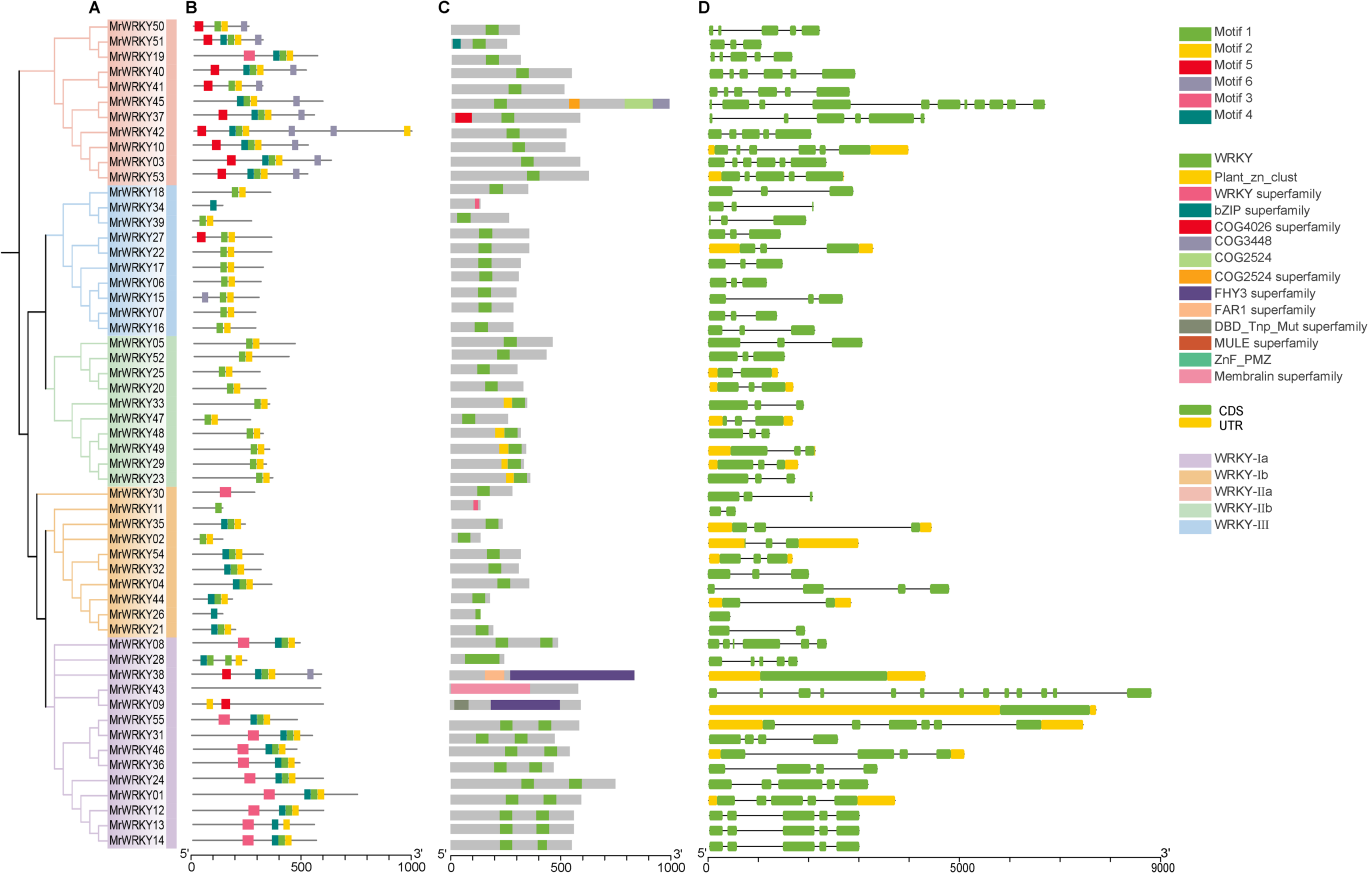
Structural composition of *WRKY* genes in bayberry. **(A)** Phylogenetic clustering of bayberry *WRKY* genes. **(B)** Distribution patterns of conserved motifs in bayberry *WRKY* genes. **(C)** Structural domain configuration of bayberry WRKY proteins. **(D)** Exon-intron structure of bayberry *WRKY* genes.

To gain deeper insights into the gene structure and phylogenetic relationships of bayberry *WRKY* genes, we conducted a detailed analysis of the structural features and conserved motifs of the *MrWRKY* family members. The evolutionary relationship of the bayberry WRKY genes was presented in **Figure 3A**. As shown in **Figure 3B**, six motifs were identified among the 55 bayberry WRKY protein sequences. Specifically, 71.43% of the WRKY-Ia subfamily contained four motifs, while approximately 60.00% of the WRKY-Ib subfamily had three motifs. Similarly, 6 (54.54%) members of the WRKY-IIa subfamily possessed five motifs, whereas 17 (80.95%) members of the WRKY-IIb and WRKY-III subfamilies (excluding MrWRKY15, MrWRKY27, and MrWRKY34) had only two motifs.

Domain analysis clearly revealed that each *WRKY* gene contained at least one WRKY domain (**Figure 3C**). As a hallmark feature of the *WRKY* gene family, this domain played a pivotal role in regulating various biological processes and was essential for gene expression and functional activity. By comparing structural differences in domains among different genes, we could further elucidate their evolutionary relationships and functional divergence.

Analysis of the *MrWRKY* gene family structure revealed that 17 (30.90%) *MrWRKY* members contained untranslated regions (UTRs) (**Figure 3D**). Specifically, 17 members possessed both 5’ and 3’ UTR exons, with exon numbers ranging from 1 to 13. Notably, *MrWRKY43* contained the highest number of exons (13), while *MrWRKY26* had only one exon and lacks introns. Within the WRKY-III subfamily, members with three exons accounted for 90.0% of the total. The *MrWRKY* gene family exhibited significant variation in gene length and intron-exon numbers, while maintaining relatively conserved gene structures within each subfamily. The similarity in gene structures and motif features among subfamilies further validated the reliability of the classification.

### 3.4 *Cis*-acting element analysis of bayberry *WRKY* genes

**Figure 4:**
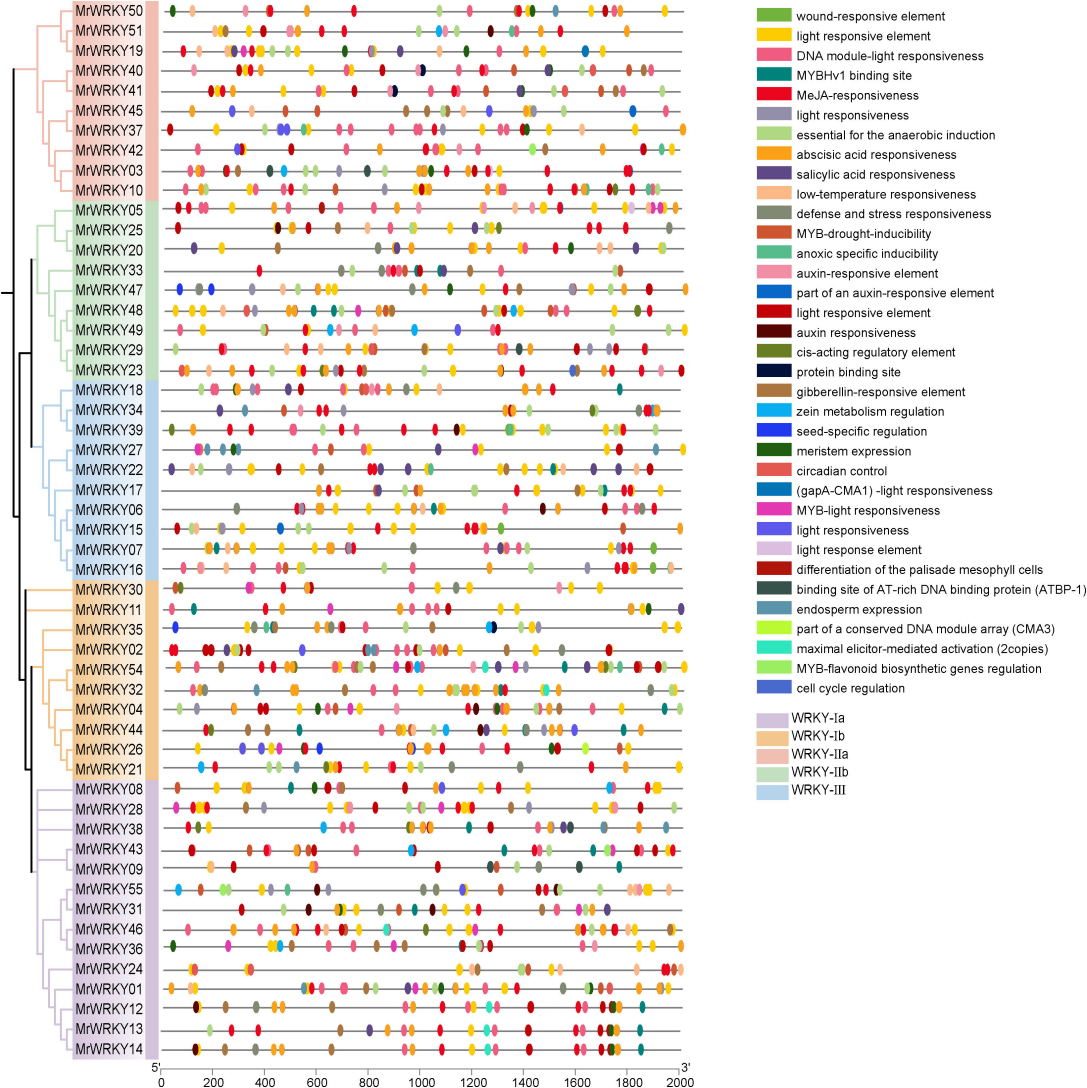
C*is*-acting elements analysis of *WRKY* genes in bayberry. Differently colored blocks represent distinct *cis*-acting elements.

To further investigate the potential functions and regulatory mechanisms of bayberry *WRKY* genes, sequences from the upstream 2000 bp promoter regions of *WRKY* genes were extracted, and an extensive *cis*-acting element analysis was conducted using the PlantCARE online tool (**Figure 4**). Analyzing the promoters of bayberry *WRKY* genes could help identify *cis*-regulatory elements and understand their regulatory networks, while also uncovering how *WRKY* genes interact with other signaling pathways in the plant. The analysis revealed that the promoter regions of two *WRKY* genes, *MrWRKY52* (a member of the WRKY-IIa subfamily) and *MrWRKY53* (a member of the WRKY-IIb subfamily), showed no detectable *cis*-acting elements.

Through a systematic analysis of the bayberry *WRKY* promoters, this study identified and classified various *cis*-regulatory elements into three major categories: hormone response elements, light response elements, and abiotic stress response elements. Notably, light response elements were widely distributed across the *WRKY* gene family. Specifically, 186 light response elements were detected in the WRKY-Ia subfamily, accounting for 44.39% of the total *cis*-acting elements in this subfamily. Similarly, 139 light response elements were found in the WRKY-Ib subfamily, representing 44.13% of its total *cis*-acting elements. Additionally, the WRKY-IIa, WRKY-IIb, and WRKY-III subfamilies contained 127, 103, and 116 light response elements, respectively, which accounted for 44.41%, 39.77%, and 39.73% of their respective total *cis*-acting elements.

In terms of hormone response elements, the WRKY-Ia subfamily exhibited the highest abundance, containing 62 methyl jasmonate (MeJA) response elements (14.79%) and 50 abscisic acid (ABA) response elements (11.93%). Analysis of stress-responsive elements revealed that the WRKY-III subfamily possessed the highest quantity, containing 60 elements in total, accounting for 20.55% of its total *cis*-acting elements repertoire. The investigation further demonstrated that the majority of *WRKY* promoters contained multiple *cis*-acting elements, with *MrWRKY54* exhibiting the maximum count of 49 *cis*-acting elements. The distribution characteristics of these *cis*- regulatory elements suggested that *WRKY* genes likely participate in responding to multiple hormone signals and environmental stresses, indicating that their expression undergoes sophisticated multi-tiered regulation during plant growth and development.

### 3.5 Collinearity analysis of bayberry *WRKY* genes

**Figure 5:**
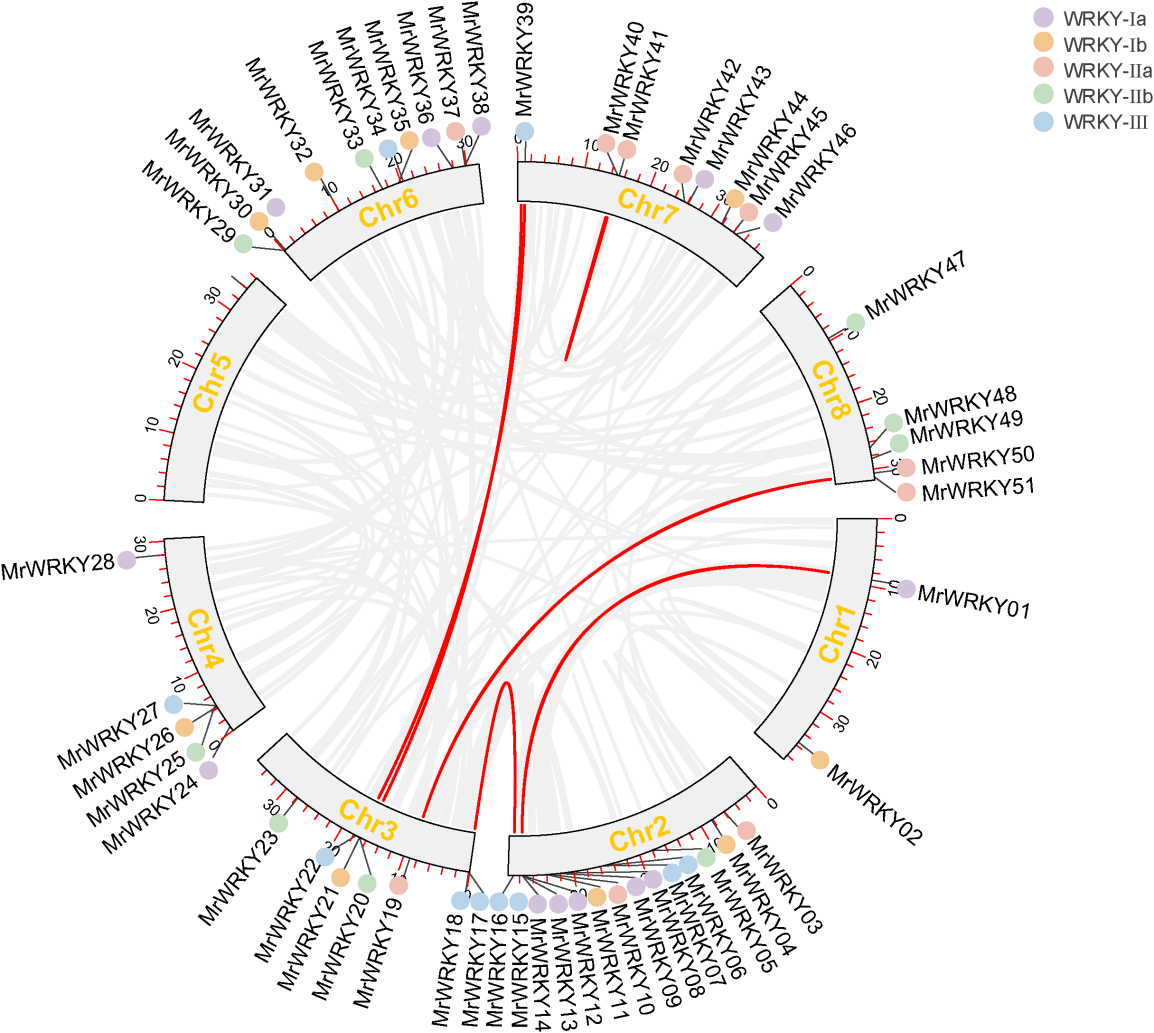
Collinearity analysis of *WRKY* gene family in bayberry.

**Figure 6:**
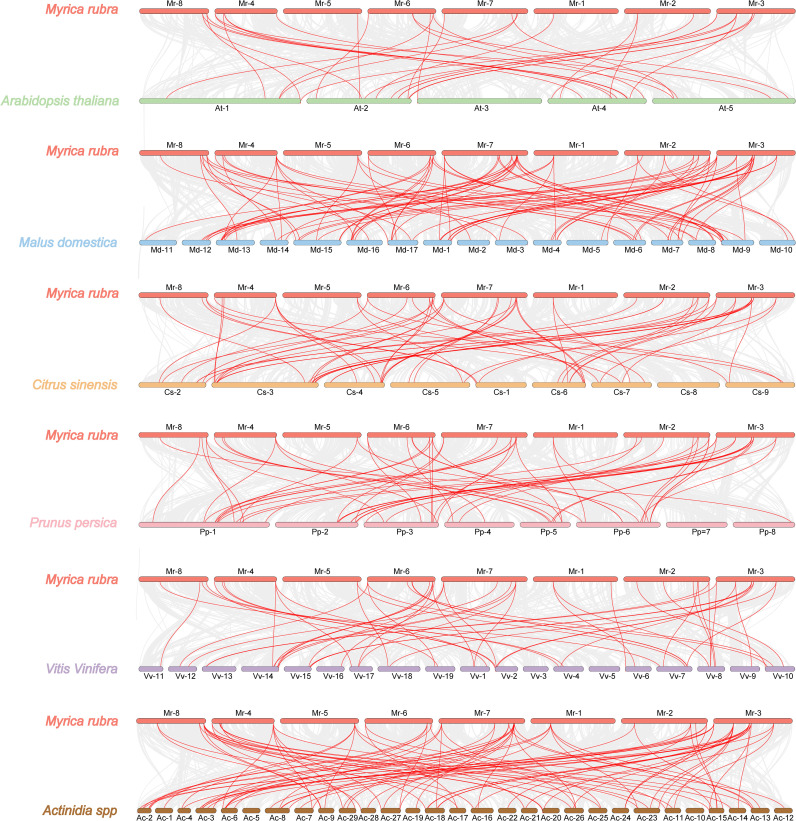
Comparative synteny analysis of *WRKY* genes among different species. Gray lines represent syntenic blocks between bayberry and other species, while red lines highlighting homologous *WRKY* gene pairs.

To investigate the evolutionary history of bayberry *WRKY* genes, collinearity analyses were conducted within and across species. As shown in **Figure 5**, the results revealed six segmental duplication events involving 12 *WRKY* genes, accounting for 21.81% of the entire *WRKY* gene family. Notably, five pairs of duplicated genes were distributed across different chromosomes, indicating that the *MrWRKY* gene family members experienced chromosomal segment duplication during evolution. Furthermore, a comparative collinearity analysis was performed between bayberry and other species, including *Arabidopsis*, apple, mandarin orange, peach, grape, and kiwifruit. As shown in **Figure 6**, 30 orthologous gene pairs were identified between bayberry and *Arabidopsis*, while apple, mandarin orange, peach, grape, and kiwifruit shared 81, 54, 52, 50, and 88 orthologous gene pairs, respectively. These findings suggested a closer evolutionary relationship between the *WRKY* gene families of bayberry and kiwifruit.

### 3.6 Expression profiling analysis of bayberry *WRKY* genes

**Figure 7:**
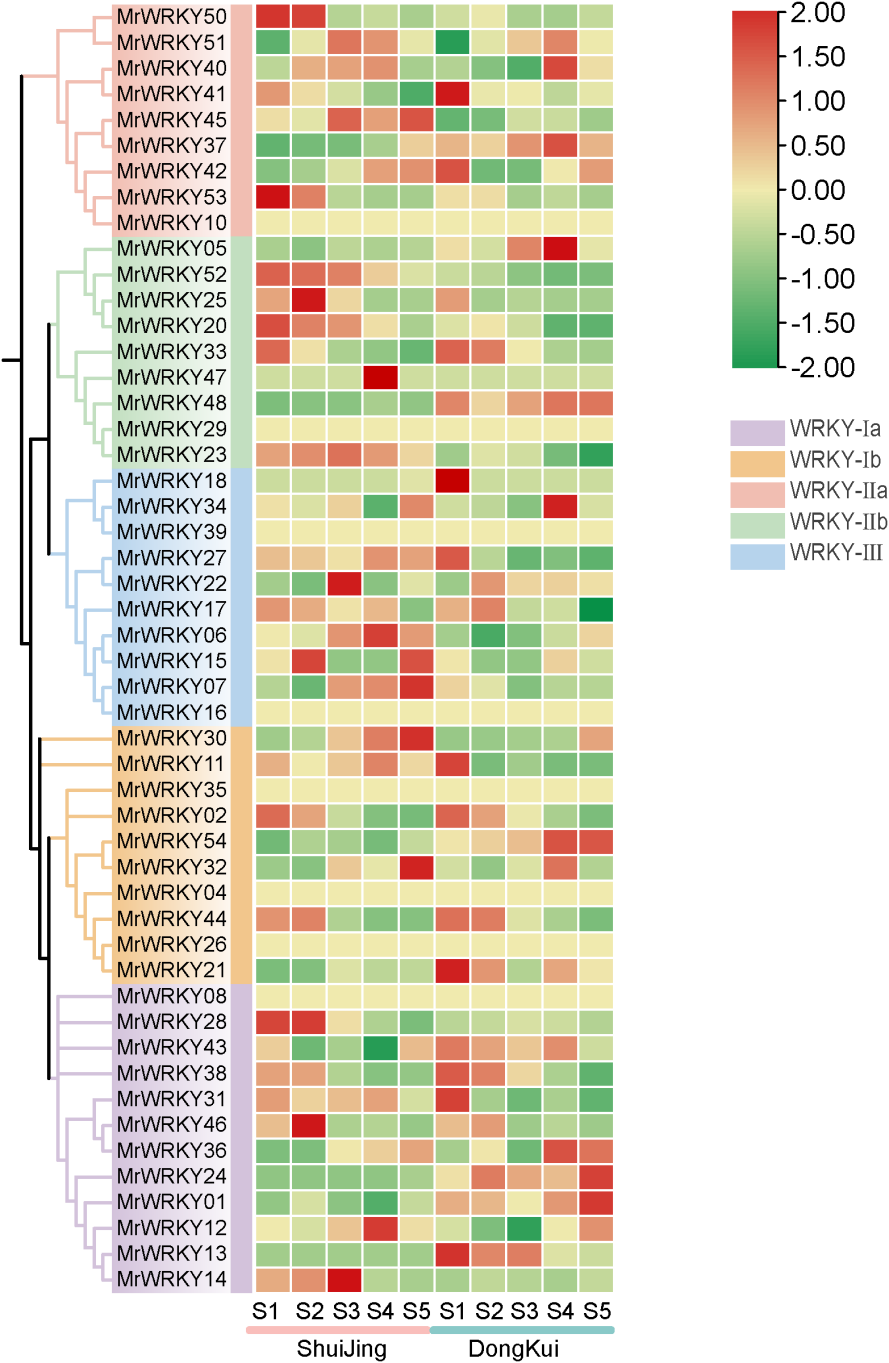
Expression analysis of *WRKY* genes during bayberry fruit development.

To investigate the expression patterns and biological functions of *WRKY* genes in bayberry fruits, two cultivars of bayberry fruits at different developmental stages were used as experimental materials (**Figure 7**). The results revealed diverse expression patterns among the bayberry *WRKY* subfamilies. In the WRKY-Ia subfamily, *MrWRKY28* was specifically highly expressed in ‘Shuijing’ at stages S1 and S2, while *MrWRKY13* was predominantly expressed during the early fruit development stage in ‘Dongkui’. *MrWRKY01* and *MrWRKY24* exhibited peak expression levels in ‘Dongkui’ during the fruit ripening stage. *MrWRKY14* exhibited higher expression levels in ‘Shuijing’ fruits compared to ‘Dongkui’. Specifically, it showed markedly elevated expression during early developmental stages (S1-S3) in ‘Shuijing’, with expression abundance progressively increasing, followed by a decline during fruit maturation (S4-S5). In contrast, the expression level in ‘Dongkui’ displayed a rapid decrease throughout the entire fruit development process.

In the WRKY-Ib subfamily, four genes showed high expression during the early fruit development stage. Among these, *MrWRKY30* and *MrWRKY32* were highly expressed in ‘Shuijing’ during fruit ripening, whereas *MrWRKY54* displayed the highest expression levels in ‘Dongkui’ at this stage. For the WRKY-IIa subfamily, *MrWRKY50* and *MrWRKY53* were both highly expressed in ‘Shuijing’ at stages S1 and S2. In the WRKY-IIb subfamily, four genes exhibited relatively high expression levels in ‘Shuijing’ during the early fruit development stage. Notably, *MrWRKY47* reached its highest expression at stage S4 in ‘Shuijing’, while the expression levels of *MrWRKY23* gradually decreased as the fruit developed.

Within the WRKY II subfamily, *MrWRKY18* and *MrWRKY27* showed high expression in ‘Dongkui’ at stage S1. In ‘Shuijing’, *MrWRKY06* and *MrWRKY07* exhibited increasing expression levels as the fruit developed. These findings demonstrate that the bayberry *WRKY* gene family exhibits a wide variety of expression patterns during fruit development and ripening.

### 3.7 Identification and functional analysis of *MrWRKY14*

**Figure 8:**
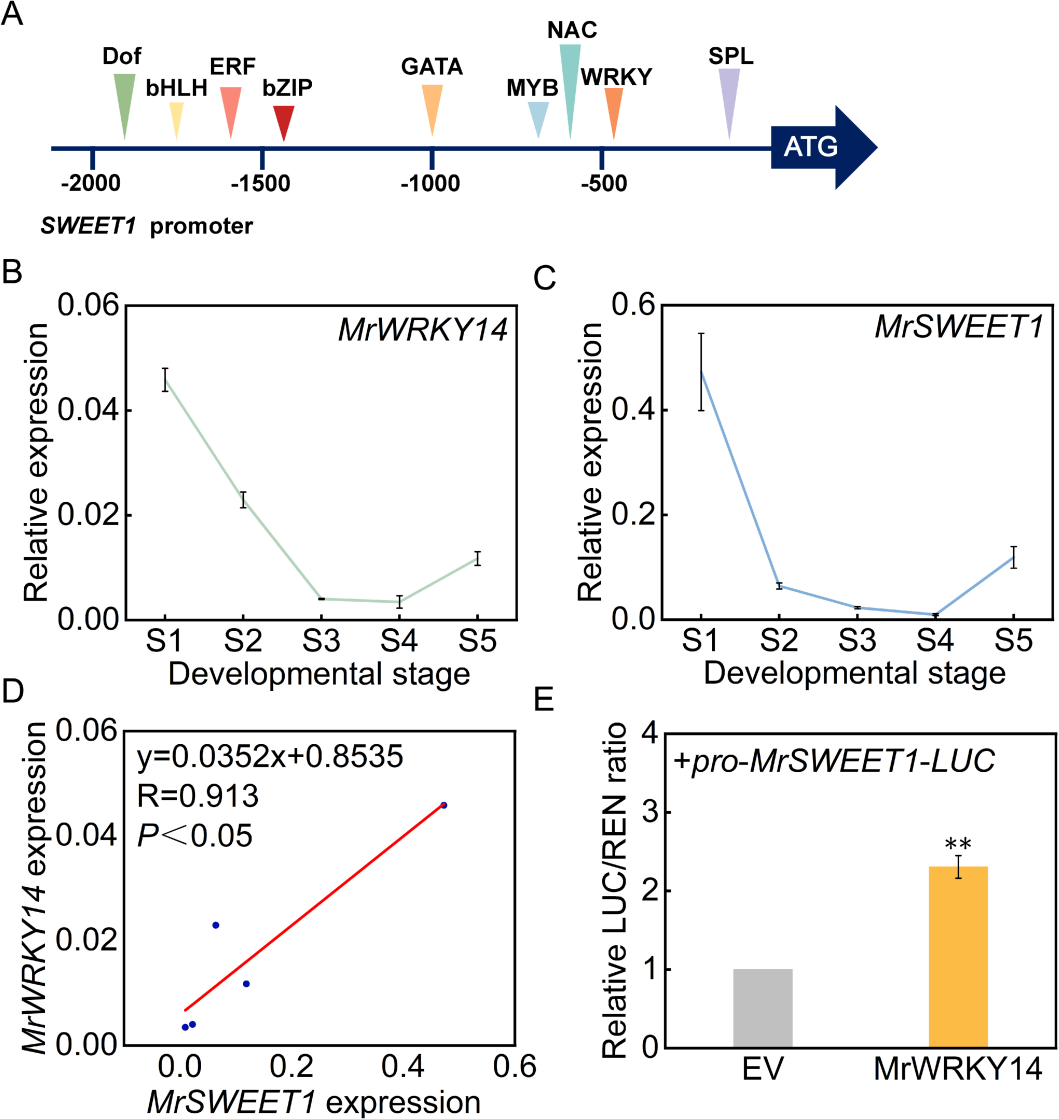
Expression pattern of *MrWRKY14* and its transcriptional regulation of *MrSWEET1* promoter. **(A)** Prediction of putative transcription factor binding sites within the *MrSWEET1* promoter region; **(B)** Expression patterns of *MrSWEET1* during bayberry fruit development; **(C)** Expression profiling of *MrWRKY14* during bayberry fruit development; **(D)** Correlation analysis between *MrWRKY14* and *MrSWEET1* expression; **(E)** Transcriptional regulation of *MrSWEET1* promoter by *MrWRKY14* using dual-luciferase reporter system. Error bars represent standard deviations calculated from three biological replicates (n=3). ** indicates significance at p < 0.01.

To further investigate the biological functions of the *WRKY* gene family members during bayberry fruit development, molecular regulatory mechanisms were studied. Based on transcriptome sequencing data, a key WRKY transcription factor member, MrWRKY14, which negatively regulated sugar accumulation in the ‘Dongkui’ fruit, was identified. Bioinformatics analysis revealed multiple potential WRKY transcription factor binding sites in the SWEET1 promoter region, suggesting a possible regulatory relationship between them (**Figure 8A**). Further analysis of the expression patterns of *MrWRKY14* and *MrSWEET1* during bayberry fruit development showed that both genes exhibited significantly downregulated expression as the fruit matured (**Figures 8B, C**).

Pearson correlation analysis demonstrated a highly significant positive correlation between the expression levels of MrSWEET1 and MrWRKY14 (R = 0.913, *P* < 0.05) (**Figure 8D**). To validate their regulatory relationship, dual-luciferase reporter assays confirmed that *MrWRKY14* significantly enhanced the transcriptional activity of the SWEET1 promoter, with a statistically significant difference compared to the empty vector control (*P* < 0.05) (**Figure 8E**). Therefore, this study demonstrated that MrWRKY14 negatively regulated fruit sugar accumulation by activating the transcriptional activity of MrSWEET1.

## 4 Discussion

Bayberry, as a precious fruit tree resource unique to China, has significant planting potential in the subtropical regions. However, the sustainable development of the bayberry industry faces numerous constraints and urgently requires breakthroughs in breeding and technological innovation (Zhang et al., 2022). WRKY transcription factors, as plant-specific regulatory proteins, play key roles in various aspects such as growth and development, stress response, and secondary metabolism (Cao et al., 2024). Systematically exploring the bayberry *WRKY* gene family and elucidating its functions not only helps to clarify the molecular regulatory mechanisms of important agronomic traits in bayberry but also accelerates the excavation of excellent genetic resources and the breeding of new varieties, which is of great strategic significance for promoting the innovative development of the bayberry industry.

This study represented the first bioinformatics-based identification of 55 WRKY transcription factor family members from bayberry (*Myrica rubra*) transcriptome data.

The number of these genes was relatively fewer compared to model plants such as *Arabidopsis* (72 genes) (Chen et al., 2017) and tomato (88 genes) (Liu et al., 2022), but it was comparable to that of grape (59 genes) (Guo et al., 2014) and peach (58 genes) (Zhong et al., 2021), reflecting the evolutionary conservation of *WRKY* gene families in woody plants. Phylogenetic analysis revealed that although the *WRKY* genes of bayberry originated from a common ancestor, they underwent significant differentiation and functional specialization during evolution, resulting in the identification of five major evolutionary clades. Within the WRKY subfamilies of bayberry, the Ia subfamily contained the largest number of members (14 genes), followed by the IIa and IIb subfamilies, each with 11 members. In contrast, the Ib and III subfamilies had the fewest members (10 genes each). Chromosomal distribution analysis of WRKY members in bayberry was uneven across 8 chromosomes, with distinct distribution patterns among different subfamilies. Notably, the highest number of members (14) was found on chromosome 2. Structural characterization demonstrated significant variations in exon-intron organization and conserved motif composition among different subfamilies of bayberry *WRKY* genes, potentially associated with their functional specificity and diversity.

Previous studies have demonstrated that *WRKY* genes in plants such as *Arabidopsis* and tomato play a crucial role in fruit ripening, regulating various fruit traits including size, color, flavor, and nutritional quality. Additionally, these genes have significant impacts on responses to both biotic and abiotic stresses (Liu et al., 2022). As a class of important transcription factors, *WRKY* genes were widely present in plants. They regulated the expression of downstream genes and participate in multiple processes of plant growth and development. During fruit development, *WRKY* genes could respond to environmental signals and changes in internal hormones, thereby controlling the expression of genes related to fruit maturation and influencing fruit characteristics (Yang et al., 2025). To further elucidate the functional and regulatory patterns of the *WRKY* gene family, a cis-element prediction of the bayberry *WRKY* gene promoters was conducted. The results revealed that bayberry *WRKY* genes exhibited responsiveness to various environmental stresses, and different subfamilies were subject to distinct regulatory mechanisms during plant growth and development. This discovery provided a new perspective for understanding the molecular mechanisms underlying fruit development and offered new insights for optimizing fruit quality.

Gene expression patterns were crucial for predicting gene functions (Jiang et al., 2025). Through comparative analysis of different developmental stages of bayberry fruit, we found that the genes *MrWRKY02*, *MrWRKY14*, *MrWRKY33*, and *MrWRKY41* exhibited high expression levels during the early fruit development stage of both cultivars. These findings suggested that these genes may play essential roles as functional genes in the development of bayberry fruit, exerting significant regulatory effects on fruit development.

Furthermore, it has been observed that *MrWRKY14* in bayberry influenced sugar accumulation through the regulation of SWEET. Functionally WRKY transcription factors associated with fruit sugar accumulation have also been identified in other fruits. For instance, in pear (*Pyrus* spp.), PuWRKY31 has been demonstrated to interact with the PuSWEET15 promoter to activate its transcription, thereby modulating sucrose accumulation (Li et al., 2020). Similarly, RsWRKY40 in radish (*Raphanus sativus*) regulated sugar accumulation by controlling RsSPS1 expression, thereby enhancing its stress tolerance (Chen et al., 2025).

In this study, a total of 55 *WRKY* family members were identified in bayberry, which were classified into five subfamilies and randomly distributed across eight chromosomes. Gene structure analysis revealed that each gene contained at least one WRKY domain, with relatively conserved gene structures maintained within each subfamily. The promoter regions of *WRKY* genes harbored multiple regulatory elements. Collinearity analysis indicated that the bayberry *WRKY* family underwent six segmental duplication events and exhibited high homology with kiwifruit. Expression pattern analysis demonstrated significant differential expression of bayberry *WRKY* genes across different cultivars and fruit developmental stages. Further investigation revealed that the WRKY family member MrWRKY14 significantly enhanced the promoter activity of *MrSWEET1*, thereby influencing sugar accumulation. These findings provided an important theoretical foundation for genome-wide identification of plant *WRKY* gene families as well as screening and characterization of functional genes.

This study represented the first systematic identification of the bayberry *WRKY* gene family, unveiling their evolutionary characteristics, structural divergence, and expression patterns. These findings not only lay a solid foundation for deeply understanding the molecular basis of bayberry’s growth and development as well as its adaptation to abiotic stresses but also provide valuable candidate genes for the exploration of excellent genetic resources and molecular design breeding in bayberry. Future research should focus on elucidating the interaction networks and regulatory mechanisms of bayberry *WRKY* genes to accelerate the advancement of bayberry functional genomics. Furthermore, cross-species comparative genomics and transcriptomic analyses would be instrumental in elucidating the evolutionary history and functional divergence of the *WRKY* gene family in flowering plants, offering new perspectives for the structural and functional studies of plant *WRKY* genes. By integrating molecular biology and bioinformatics approaches, our understanding of the functions of *WRKY* genes in bayberry and other plants can be significantly enhanced in the future. This comprehensive understanding will provide a robust theoretical foundation and technological support for bayberry variety improvement and industrial upgrading.

## Data Availability

The datasets presented in this study can be found in online repositories. The names of the repository/repositories and accession number(s) can be found in the article/**Supplementary Material**.

## Supplementary material

Supplementary Table 1 Physicochemical properties analysis of WRKY proteins in bayberry.
